# Comparison of the efficacy of cochlear implantation and stapes surgery in far advanced otosclerosis: a meta-analysis study

**DOI:** 10.1007/s00405-022-07449-w

**Published:** 2022-06-10

**Authors:** Ahmed Abdelmoneim Teaima, Abdelhamid Abdelhamid Elnashar, Ehab Kamal Hakim, Hanaa Sabry Hadaey

**Affiliations:** Otorhinolaryngology Department, Faculty of Medicine, Ain Shams University, Abbassia Square, Ramses Street, Cairo, 11591 Egypt

**Keywords:** Otosclerosis, Stapedectomy, Stapedotomy, Cochlear implantation

## Abstract

**Objective:**

This study is to compare the hearing outcomes and complications of stapes surgery and cochlear implantation (CI) in patients with far-advanced otosclerosis (FAO).

**Data sources:**

A comprehensive electronic search of PubMed/MEDLINE, Scopus, Web of science and Cochrane Library was conducted in June 2021 for articles in the literature till this year.

**Study selection:**

Studies are published in English language, conducted on human subjects, concerned with comparison of CI and stapes surgery in the management of FAO, not Laboratory study and not Opinion study. The current review followed the guidelines of preferred reporting items for systematic reviews and meta-analysis statement 2009 (PRISMA).

**Data extraction:**

Twenty-six studies were included with 334 patients in CI group and 241 patients in stapes surgery group. Comparison between both groups was done in terms of postoperative complications, audiological outcomes, rete of revision surgery and patients’ satisfaction rate.

**Results:**

Postoperative complications rate was significantly lower in CI (13.6%) than stapes surgery (18.6%). CI had a significantly lower rate of revision surgery (8.1%) than stapes surgery (16.4%). CI had a better mean for pure tone average (29.1 dB) than stapedectomy (52.3 dB) while stapes surgery had a higher mean for recognition of monosyllables and disyllables than CI. CI had significantly higher satisfaction rate than stapes surgery.

**Conclusion:**

Both Stapes surgery and CI are reliable treatment options for FAO with close success rates. Statistics of CI are greater than stapes surgery and CI has a consistent improvement in audiometric outcomes in comparison to stapes surgery.

## Introduction

Otosclerosis is a disorder of the labyrinthine capsule, formed of bone resorption then reparative deposition of new, immature sclerotic bone [1]*.* Otosclerotic foci may extend deeper into the labyrinth, resulting in retrofenestral otosclerosis and severe mixed hearing loss which is known as far-advanced otosclerosis (FAO) [2]. FAO was first defined by House in 1961 as air conduction (AC) threshold by 85 dB in otosclerosis patients. There is no universally accepted definition for advanced otosclerosis. Calmels et al. defined FAO audiologically as decrease dissyllabic words less than 30% of the speech discrimination (SD) score at 70 dB [3]. There are no standard guidelines for management of FAO. The intervention options include stapes surgery and hearing aid, or cochlear implantation (CI) [2, 4]. Each has its advantages, disadvantages, results and complications [5]. So, the objective of our study is to compare the hearing outcomes and complications of stapes surgery and cochlear implantation in patients with far-advanced otosclerosis.

## Patients and methods

### Literature search

**Fig. 1:**
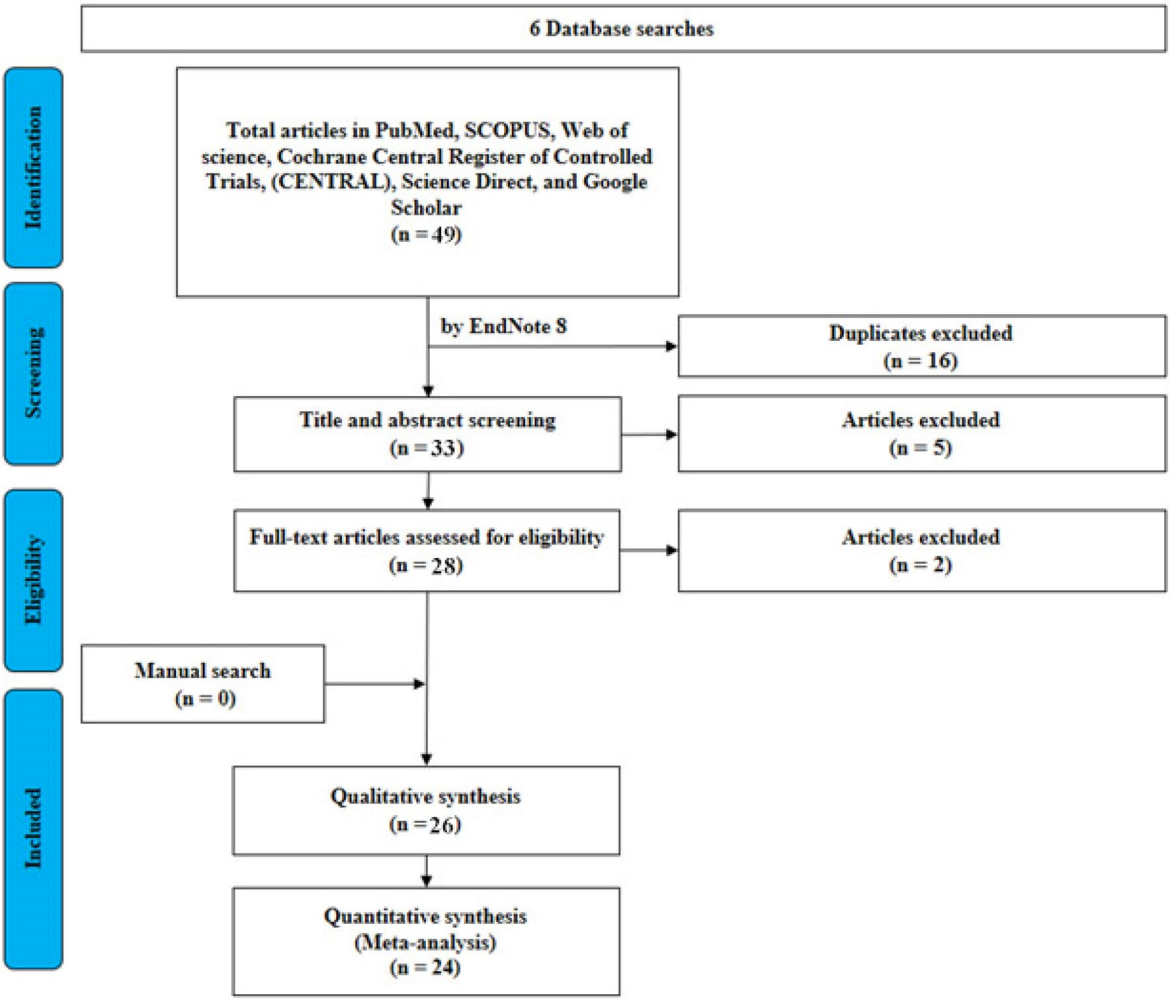
PRISMA flow diagram of the search and review process

A comprehensive electronic search of PubMed/MEDLINE, Scopus, Web of Science and Cochrane Library was conducted in June 2021 for articles in the literature till this year. Only English studies concerning stapes surgery or cochlear implantation in FAO were included using a combination of the following key words: far-advanced otosclerosis, stapedectomy, stapes surgery, stapedotomy, cochlear implantation, cochlear implant. Article selection and screening proceeded according to the search strategy based on Preferred Reporting Items for Systematic Reviews and Meta-analysis criteria Fig. 1. Cited references in the screened articles were also assessed for relevance to maximize sensitivity. 312 articles were yielded, from which 51 articles met our criteria. After duplicates removal, 35 articles were screened in title/abstract screening, while 30 articles were screened in full text screening for inclusion. Finally, 26 articles were included. Detailed characteristics of the included studies are shown in Table 1.

**Table 1:** Characteristics table for patients in the included articles

Reference ID	Type of study	Sample size	Follow-up period (years)	Type of surgery	Age (years) [mean (SD)]	Sex (Female) *n* (%)	QA tool
Castillo/2014/Spain [6]	Prospective Cohort	17	0.5, 1, 2, 3, 5	CI	55.6	13 (76.5)	Good
Lopez/2006/Spain [7]	Prospective Cohort	30	5.8	CI	51 (41)	24 (80)	Good
Dumas/2018/USA [8]	Retrospective Cohort	35	1	CI	59 (8)	16 (45.7)	Good
Psillas/2007/Greece [4]	Retrospective Cohort	5	NA	CI	60.2	3 (60)	
Luca/2021/Italy [9]	Retrospective Cohort	11	0.5, 1, 3	Stapedectomy	69.5	5 (45.5)	Good
Calmels/2007/France [3]	Retrospective Cohort	7	2 months	Stapedectomy	70.9	3 (42.9)	Good
		7		CI	63.9	5 (71.4)	Fair
Redfors/2011/Sweden [10]	Retrospective Cohort	65	30	Stapedectomy	NA	NA	Good
Dejaco/2018/Austria [11]	Case report	1	31 days	CI	NA	NA	Poor
Frattali/1993/USA [12]	Retrospective Cohort	9	NA	Stapedectomy	NA	NA	Fair
Ghonim/1997/Egypt [13]	Retrospective Cohort	8	NA	Stapedectomy	49 (4.75)	3 (37.5)	Fair
Glasscock/1996/USA [14]	Retrospective Cohort	15	0.25, 1	Stapedectomy	62	8 (53.3)	Good
Heining /2017/UK [5]	Retrospective Cohort	28	NA	Stapedectomy	NA	NA	Fair
Lurato/1985/Italy [15]	Retrospective Cohort	34	1	Stapedectomy	NA	NA	Good
Kabbara/2015/France [16]	Retrospective Cohort	32	1	Stapedectomy	59 (11.9)	NA	Good
		34	1	CI			
Khalifa/1998/Egypt [17]	Retrospective Cohort	8	NA	Stapedectomy	61	5 (62.5)	Fair
Lachance/2012/Canada [18]	Retrospective Cohort	16	1	Stapedectomy	NA	NA	Good
Lovato/2020/Italy [19]	Retrospective Cohort	5	1	CI	59.6	3 (60)	Good
Marshall/2005/Canada [20]	Retrospective Cohort	25	0.5, 1	CI	4.7	NA	Good
Mosniera/2007/France [21]	Retrospective Cohort	16	0.5, 8	CI	61	9 (56.3)	Good
Rotteveel/2004/UK [22]	Retrospective Cohort	53	NA	CI	NA	NA	Fair
Rotteveel/2010/UK (23)	Retrospective Cohort	53	NA	CI	NA	NA	Fair
Ruckenstein2001/USA (24)	Retrospective Cohort	8	1	CI	62	2 (25)	Good
Sainz/2009/Spain [25]	Prospective Cohort	15	6	CI	32.6 (8.6)	NA	Good
Semaan/2012/USA [26]	Retrospective Cohort	30	1	CI	72 (5)	16 (53)	Good
Bajin/2020/Turkey [27]	Retrospective Cohort	8	2.3	Stapedectomy	56	7 (36.8)	Good
		13		CI			
Vashishth/2017/Italy (28)	Retrospective Cohort	38	4	CI	59.72	11 (29)	Good

### Quality assessment

The quality of relevant studies was assessed using NIH quality assessment tool for observational cohort studies. (“Study Quality Assessment Tools | National Heart, Lung, and Blood Institute (NHLBI),” 2019) Regarding cohort studies, each study was given a score out of 14 based on answering each question (Yes = 1, No = 0, NA = 0). A score of 10–14 indicated a good quality article, 5–9 for fair, and 1–4 for poor quality article. Regarding case series studies, total evaluation score was 9, a score from 7 to 9 indicated good quality article, whereas score from 4 to 6 for fair, and 1–3 for poor quality article. Regarding quality assessment, from 26 studies, 18 were evaluated with good quality, 7 were fair, and 1 was poor.

### Statistical analysis

We made pairwise meta-analysis of our outcomes using Comprehensive Meta-Analysis software (CMA version 3.9). Odds ratio (OR) with the corresponding 95% confidence intervals (95% CI) was also be calculated for categorical data. While dichotomous variables with one group were assessed by event rate and its corresponding 95% CI. A fixed-effects model was used when there was no heterogeneity. Heterogeneity was assessed with Q statistics and I2-test considering it significant with I2 value > 50% or *P*-value < 0.10.

## Results

### Postoperative complications

**Fig. 2:**
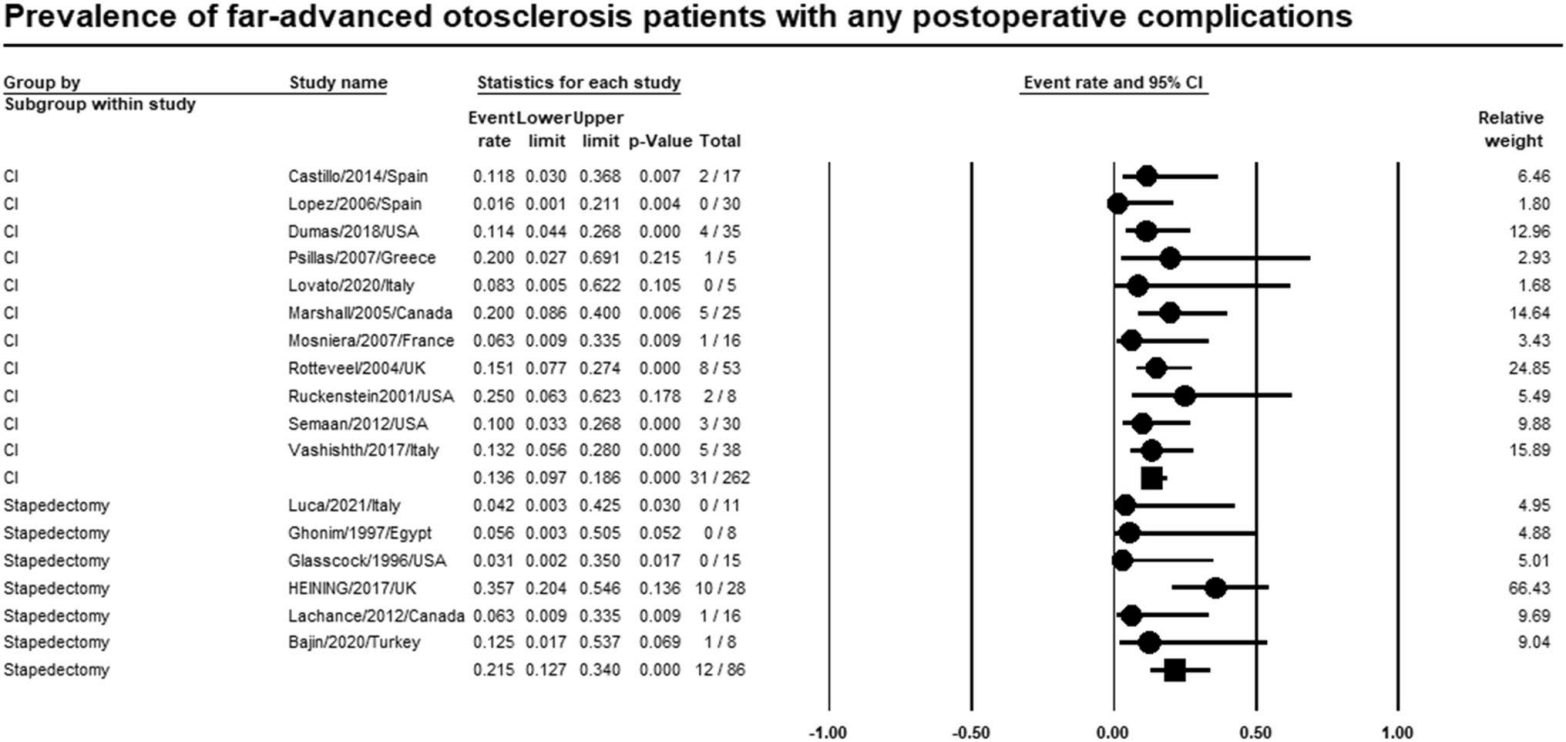
Meta-analysis for any postoperative complications rate

Meta-analysis of relevant studies showed that CI had significant lower rate of any postoperative complications in patients with far-advanced otosclerosis [Event rate = 13.6%, 95% CI (9.7–18.6%), *P*-value < 0.001]. While any postoperative complications rate of stapedectomy was [Event rate = 21.5, 95% CI (12.7–34%), *P*-value < 0.001] (Fig. 2).

### Difficult access to area of cochleostomy

**Fig. 3:**
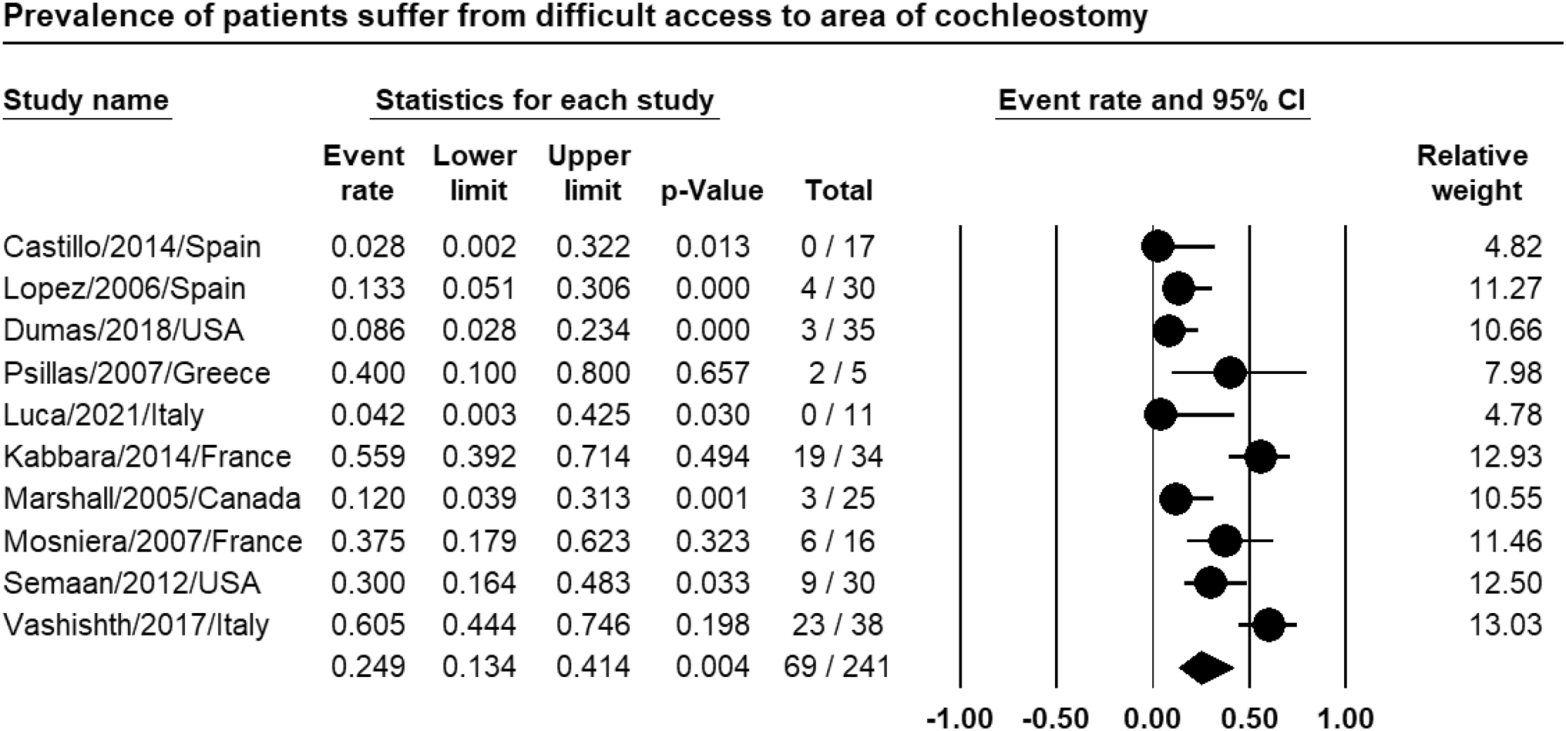
Meta-analysis for difficult access to area of cochleostomy rate in CI

Meta-analysis of relevant studies showed that CI had significant low rate of difficult access to area of cochleostomy in patients with far-advanced otosclerosis [Event rate = 24.9%, 95% CI (13.4–41.4%), *P*-value = 0.004] (Fig. 3).

### Difficult insertion of electrode bundle

**Fig. 4:**
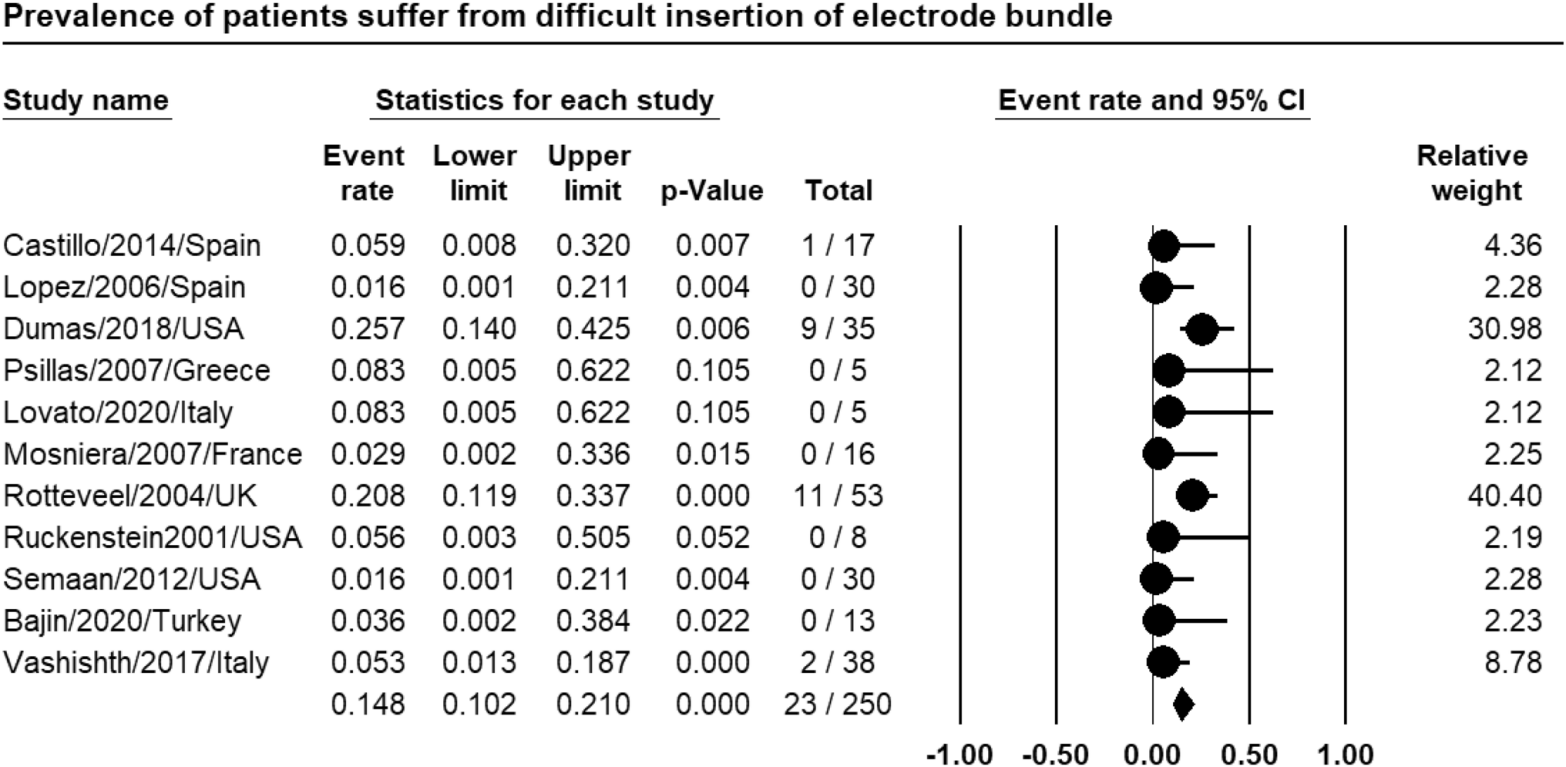
Meta-analysis for difficult insertion of electrode bundle rate in CI

Meta-analysis of relevant studies showed that CI had significant low rate of difficult insertion of electrode bundle in patients with far-advanced otosclerosis [Event rate = 14.8%, 95% CI (10.2–21%), *P*-value < 0.001] (Fig. 4).

### Dysgeusia

**Fig. 5:**
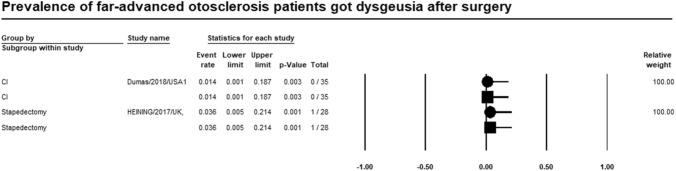
Meta-analysis for dysgeusia rate

Meta-analysis of relevant studies showed that CI had significant lower rate of dysgeusia in patients with far-advanced otosclerosis [Event rate = 1.4%, 95% CI (0.1–18.7%), *P*-value = 0.003]. While dysgeusia rate of stapedectomy was [Event rate = 3.6%, 95% CI (0.5–21.4%), *P*-value = 0.001] (Fig. 5).

### Tinnitus

**Fig. 6:**
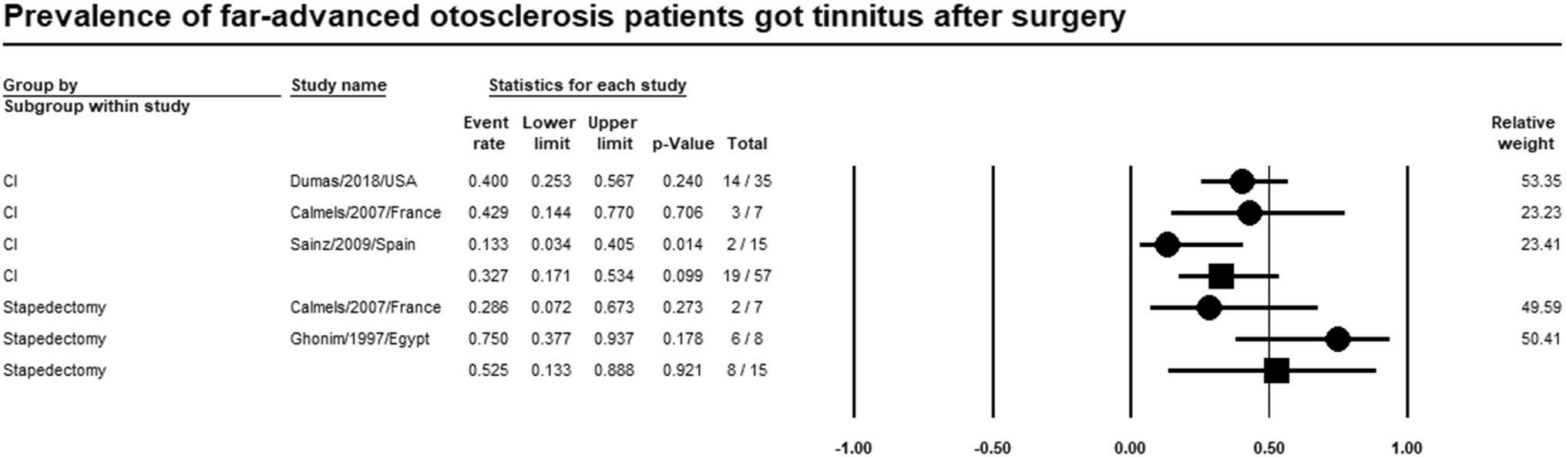
Meta-analysis for tinnitus rate

Meta-analysis of relevant studies showed that CI had lower rate of tinnitus in patients with far-advanced otosclerosis [Event rate = 32.7%, 95% CI (17.1–53.4%), *P*-value = 0.099]. While tinnitus rate of stapedectomy was [Event rate = 52.5%, 95% CI (13.3–88.8%), *P*-value = 0.001] (Fig. 6).

### Vertigo

**Fig. 7:**
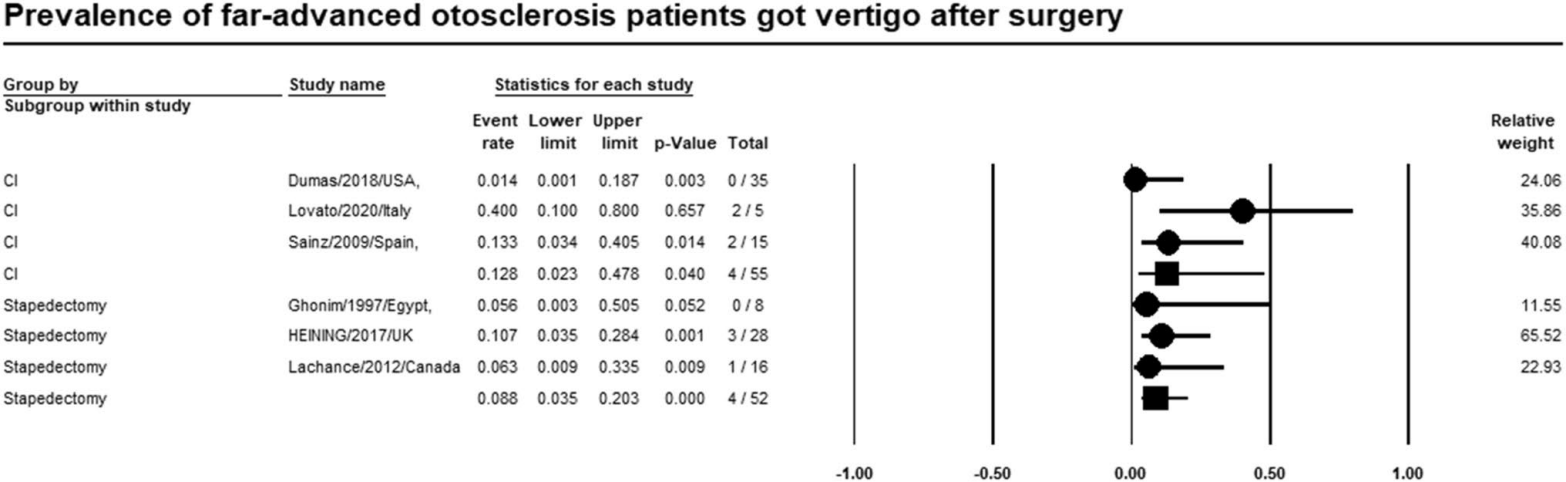
Meta-analysis for vertigo rate

Meta-analysis of relevant studies showed that stapedectomy had significant lower rate of vertigo in patients with far-advanced otosclerosis [Event rate = 8.8%, 95% CI (3.5–20.3%), *P*-value < 0.001]. While vertigo rate of CI was [Event rate = 12.8%, 95% CI (2.3–47.8%), *P*-value = 0.040] (Fig. 7).

### Facial electrical stimulation

**Fig. 8:**
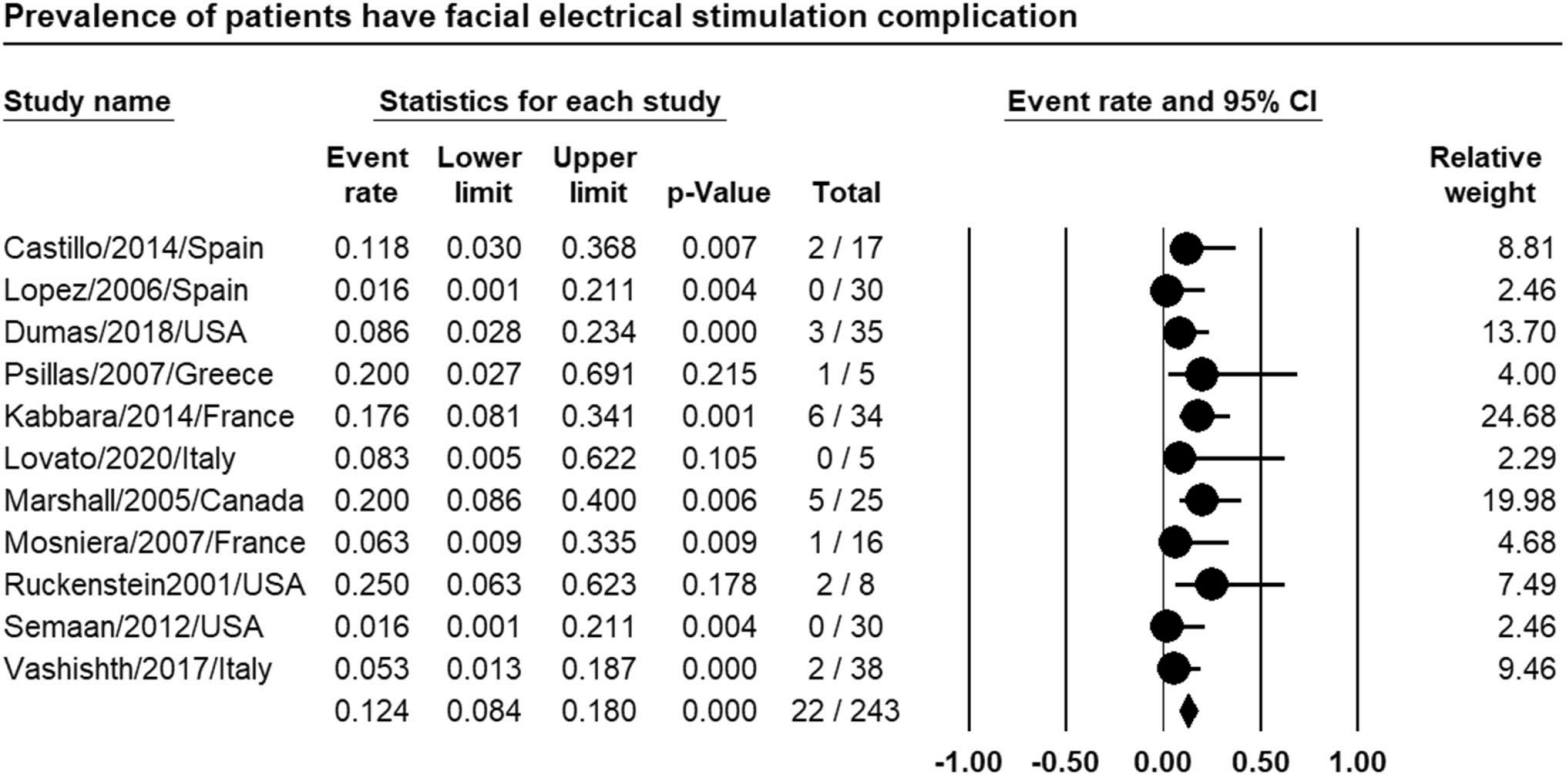
Meta-analysis for facial electrical stimulation rate in CI

Meta-analysis of relevant studies showed that CI had a significant low rate of facial electrical stimulation in patients with far-advanced otosclerosis [Event rate = 12.4%, 95% CI (8.4–18%), *P*-value < 0.001] (Fig. 8).

### Postoperative hearing loss

**Fig. 9:**
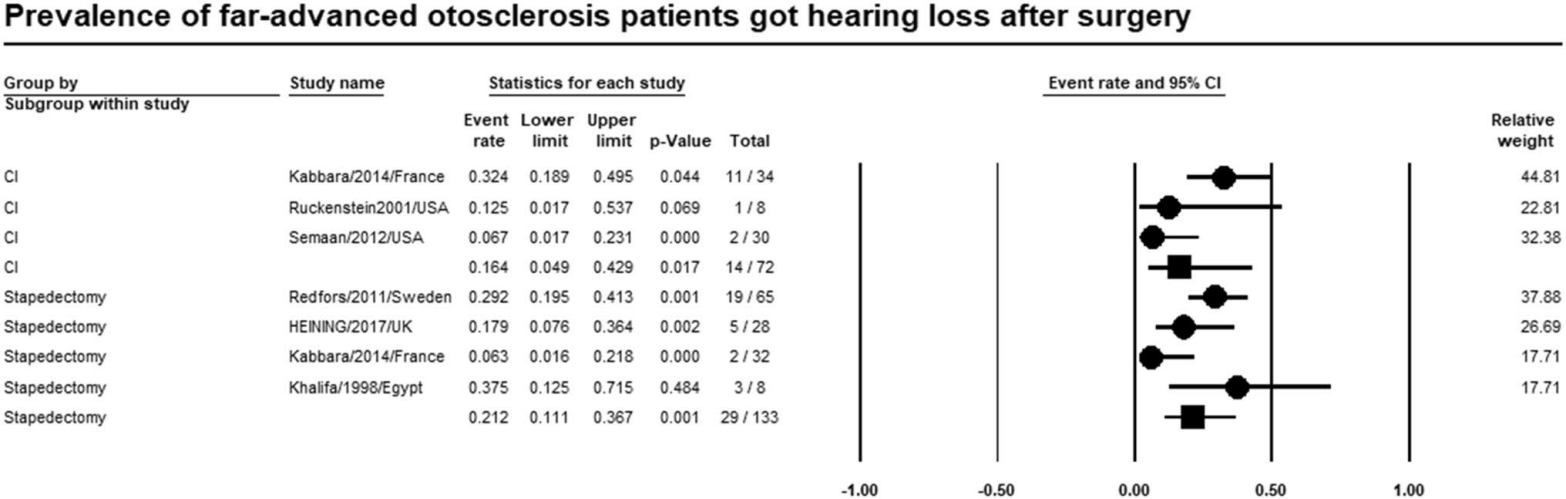
Meta-analysis for hearing loss rate after surgery

Meta-analysis of relevant studies showed that CI had significant lower rate of hearing loss after surgery in patients with far-advanced otosclerosis [Event rate = 16.4%, 95% CI (4.9–42.9%), *P*-value = 0.017]. While hearing loss rate after surgery of stapedectomy was [Event rate = 21.2%, 95% CI (11.1–36.7%), *P*-value < 0.001] (Fig. 9).

### Revision surgery

**Fig. 10:**
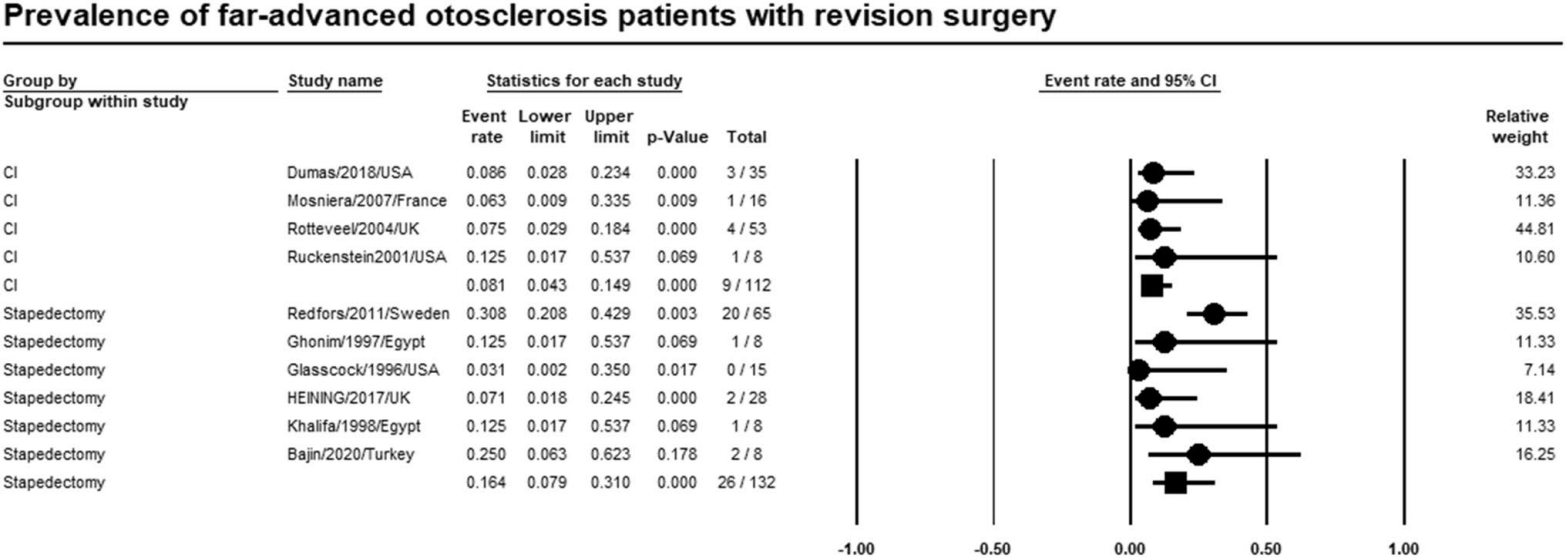
Meta-analysis for revision surgery rate

Meta-analysis of relevant studies showed that CI had a significant lower rate of revision surgery rate in patients with far-advanced otosclerosis [Event rate = 8.1%, 95% CI (4.3–14.9%), *P*-value < 0.001]. While revision surgery rate of stapedectomy was [Event rate = 16.4%, 95% CI (7.9–31%), *P*-value < 0.001] (Fig. 10).

### Recognition of monosyllables

**Fig. 11:**
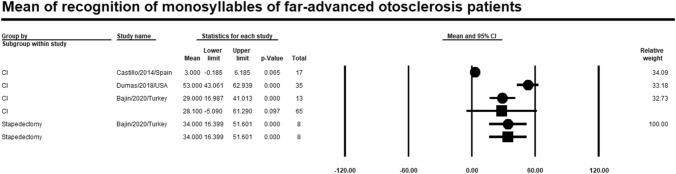
Meta-analysis for mean recognition of monosyllables

Meta-analysis of relevant studies showed that stapedectomy had a higher significant mean for recognition of monosyllables in patients with far-advanced otosclerosis [Mean = 34%, 95% CI (16.4–51.6%), *P*-value < 0.001]. While mean recognition of monosyllables of CI was [Mean = 28.1%, 95% CI (5.1–61.3%), *P*-value = 0.097] (Fig. 11).

### Recognition of disyllables

**Fig. 12:**
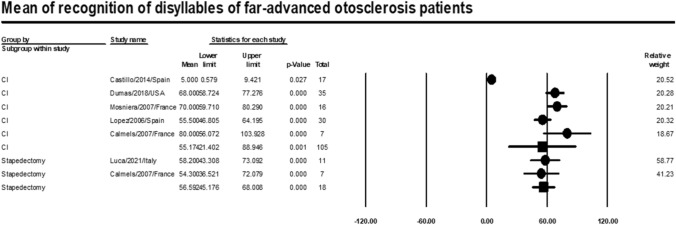
Meta-analysis for mean recognition of disyllables

Meta-analysis of relevant studies showed that stapedectomy had a higher significant mean for recognition of disyllables in patients with far-advanced otosclerosis [Mean = 56.6%, 95% CI (45.2–68%), *P*-value < 0.001]. While mean recognition of disyllables of CI was [Mean = 55.2%, 95% CI (21.4–89%), *P*-value = 0.001] (Fig. 12).

### Recognition of phrases

**Fig. 13:**
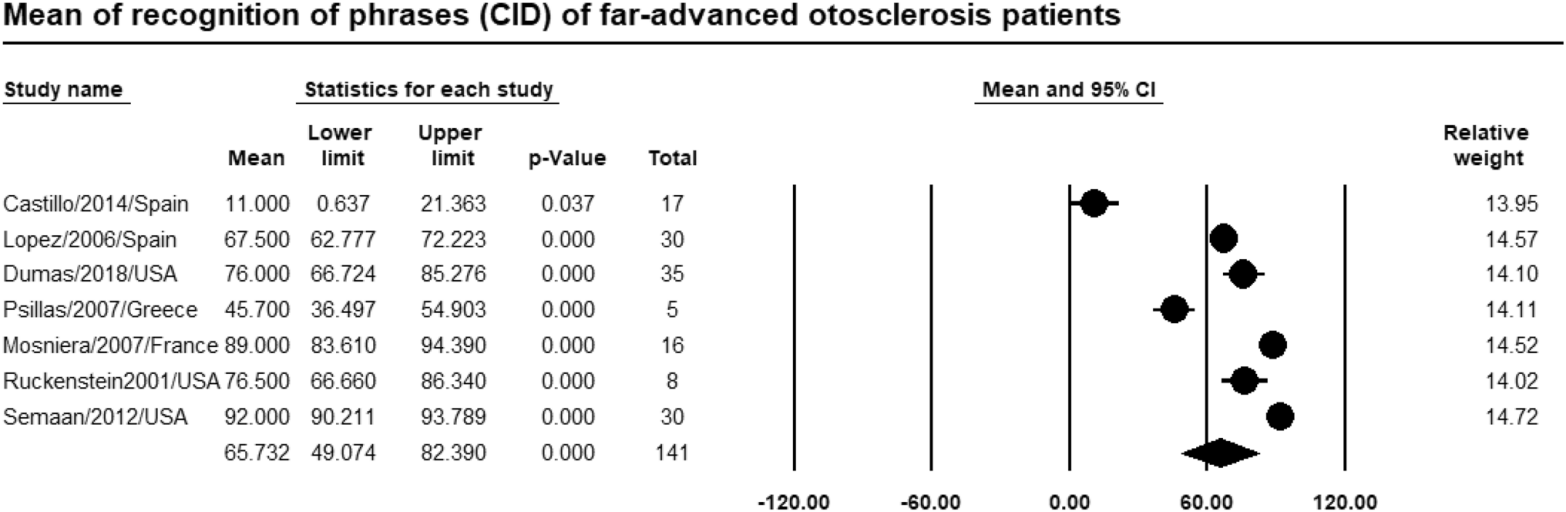
Meta-analysis for mean recognition of phrases

Meta-analysis of relevant studies showed that CI had a high significant mean for recognition of phrases in patients with far-advanced otosclerosis [Mean = 65.7%, 95% CI (49.1–82.4%), *P*-value < 0.001] (Fig. 13).

### Postoperative pure tone average

**Fig. 14:**
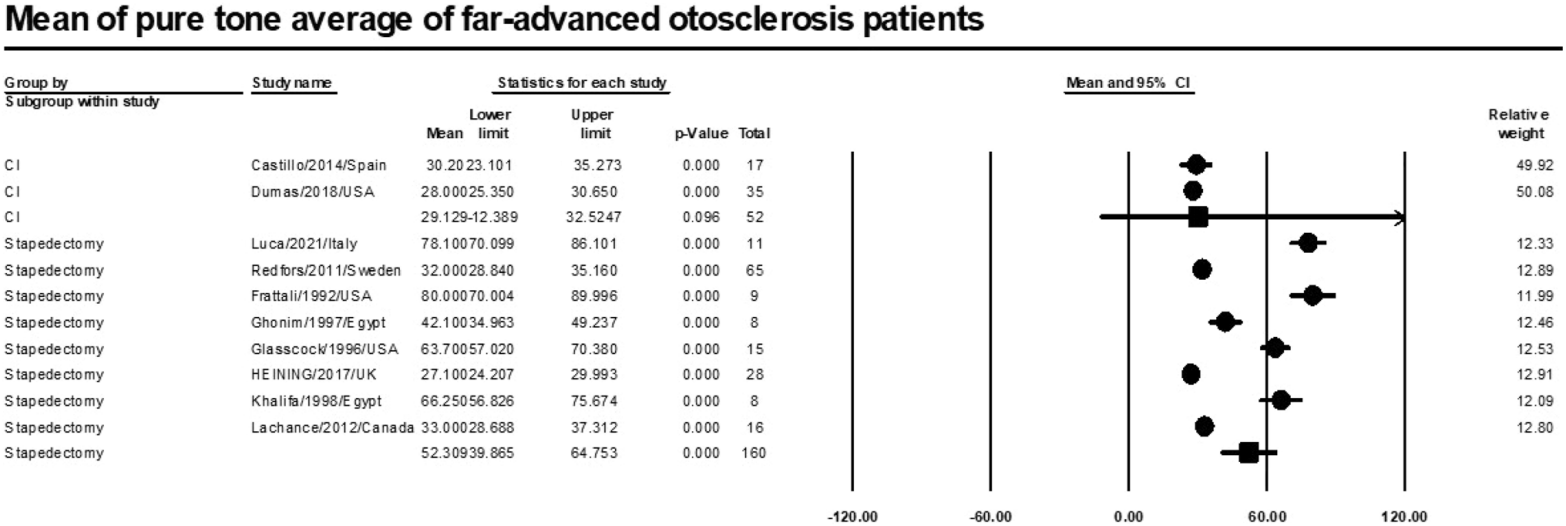
Meta-analysis for mean pure tone average

Meta-analysis of relevant studies showed that CI had a better mean for pure tone average in patients with far-advanced otosclerosis [Mean = 29.1 dB CI (29.1–32.5), *P*-value = 0.096]. While mean pure tone average of stapedectomy was [Mean = 52.3 dB CI (39.9–64.8), *P*-value < 0.001] (Fig. 14).

### Speech reception threshold

**Fig. 15:**
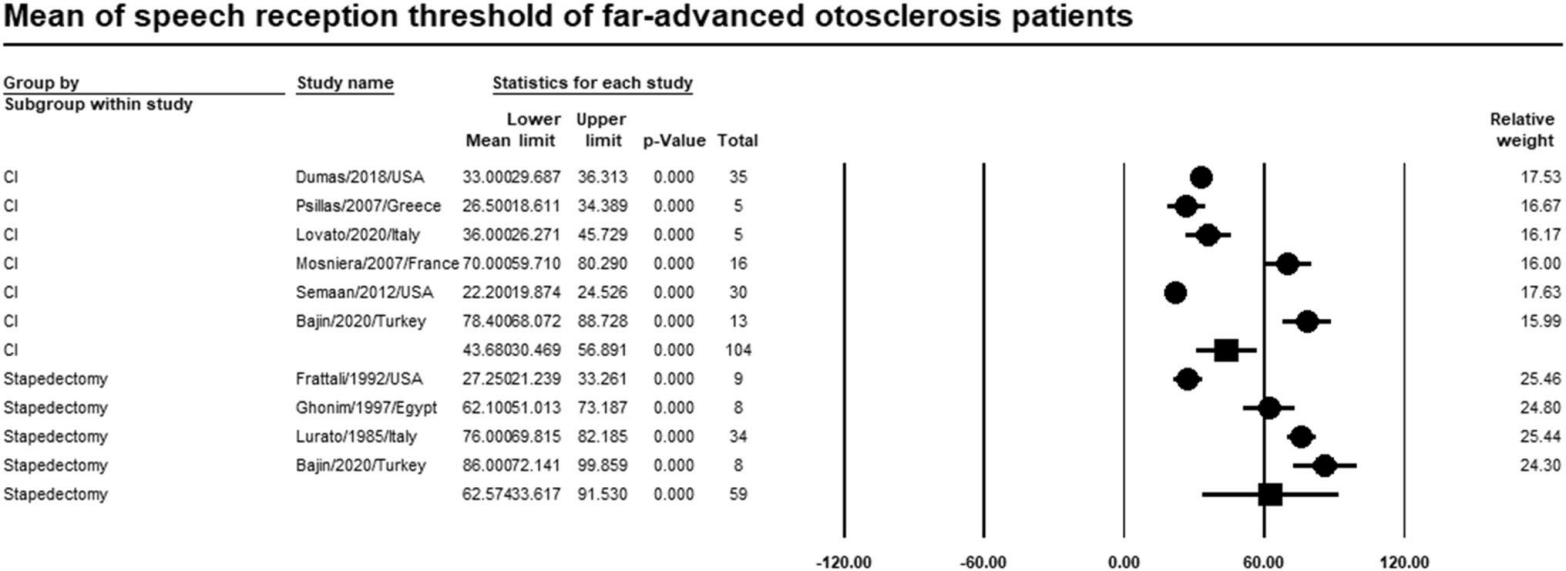
Meta-analysis for mean speech reception threshold

Meta-analysis of relevant studies showed that stapedectomy had a higher significant mean for speech reception threshold in patients with far-advanced otosclerosis [Mean = 62.6 dB, CI (33.6–91.5%), *P*-value < 0.001]. While mean speech reception threshold of CI was [Mean = 43.7 dB, CI (30.5–56.9%), *P*-value < 0.001] (Fig. 15).

### Satisfaction rate

**Fig. 16:**
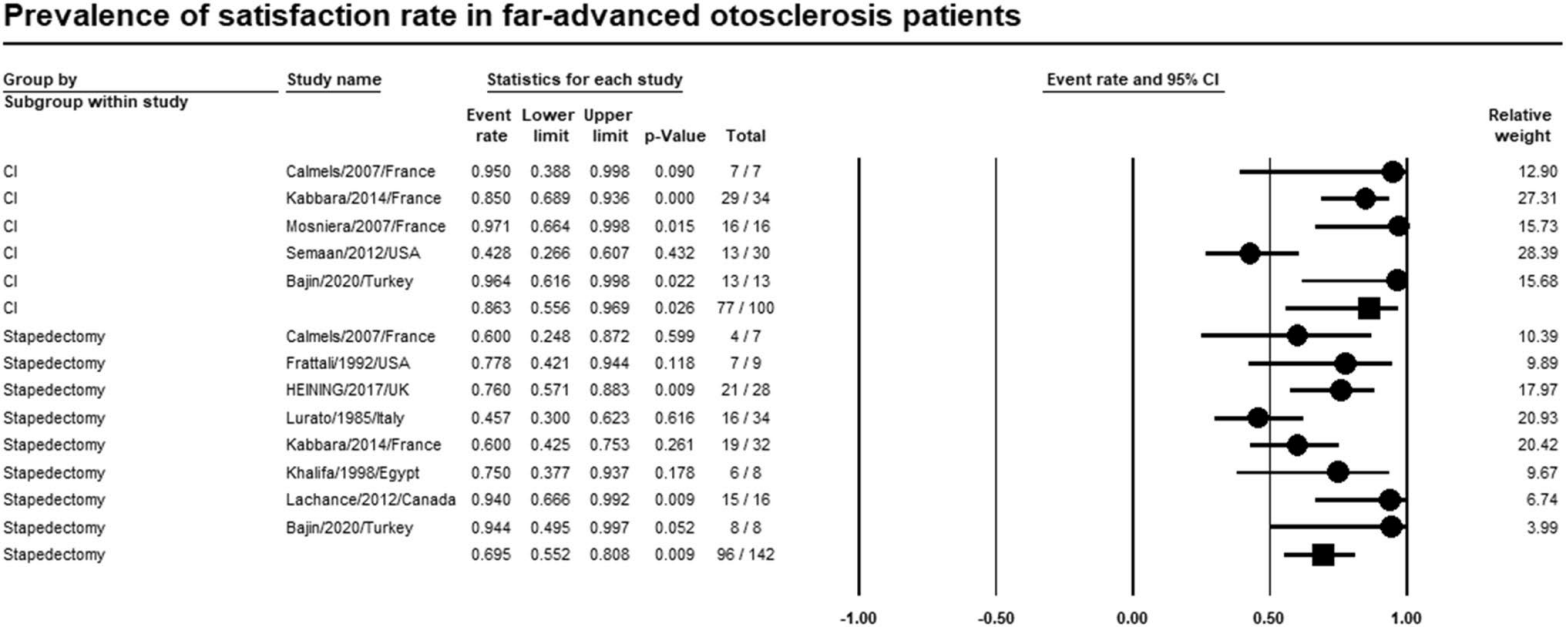
Meta-analysis for satisfaction rate

Meta-analyses of relevant studies showed that CI was significantly higher satisfaction rate than stapedectomy in patients with far-advanced otosclerosis [Event rate = 86.3%, 95% CI (55.6–96.9%), *P*-value = 0.026]. While satisfaction rate of stapedectomy was [Event rate = 69.5%, 95% CI (55.2–80.8%), *P*-value = 0.009] (Fig. 16).

## Discussion

The management of FAO has evolved over the past 20 years with the availability of CI beside stapes surgery. Each procedure has its advantages and disadvantages. Many factors may affect the choice of the management plan like the contralateral ear hearing level, duration of hearing loss, economic issues, complication rates, patient preference [16, 27]. So, this study is primarily concerned with the comparison of CI and stapes surgery in patients with FAO through metanalysis of relevant studies.

Twenty-six studies were included in the metanalysis. Meta-analysis of relevant studies showed that postoperative complications rate was significantly lower in CI (13.6%) than stapes surgery (18.6%) in patients with far-advanced otosclerosis. Dysgeusia was lower in CI (1.4%) than stapes surgery (3.6%). Tinnitus was lower in CI (32.7%) than stapes surgery (52.2%). Vertigo was lower in stapes surgery (8.8%) than in CI (12.8%). Hearing loss was lower in CI (16.4%) than stapes surgery (21.1%). CI had a significantly lower rate of revision surgery (8.1%) than stapes surgery (16.4%) in patients with far-advanced otosclerosis.

Sainz et al. and Semaan et al. found tinnitus 13.3%, 6.7% in patients with FAO after CI [25, 26]. Bajin et al. reported perilymph oozing led to total sensorineural hearing loss in one patient after stapes surgery which needed CI after that [27]. According to Heining et al. 7% of FAO patients needed revision of stapes surgery [5]. In Baijin’s study, CI was done in thirteen patients with FAO, seven of them had prior failed stapes surgeries [27].

Meta-analysis showed that CI had low rate of difficult access to area of cochleostomy (24.9%), significantly low rate of difficult insertion of electrode bundle (14.8%), low rate of facial electrical stimulation (12.4%) in patients with far-advanced otosclerosis.

Castillo et al. had one case of cochlear ossification out of seventeen patients with FAO who were managed by CI. The long-term results were similar to the other patients in spite of partial insertion [6]. Marshall et al. stated that FNS occurred in 17% of the patients with FAO after CI in comparison to control group. Management required deactivation of one or more implant electrodes [20]. Some studies showed rate of facial electrical stimulation in CI in FAO as 7% to 75%, with an average of 20%. Rotteveel et al. reported problems in electrode insertion during CI in FAO in 10 of 53 patients (3 misplacement, 7 electrode partial insertion) [22]. Semaan et al. showed that complete electrode insertion in CI was done in all the thirty-four patients with FAO of their study [26].

Our study showed that CI had a better mean for pure tone average (29.1 dB) than stapes surgery (52.3 dB). CI had a high significant mean for recognition of phrases (65.7%). Stapes surgery had a higher mean for recognition of monosyllables and disyllables in patients with far advanced otosclerosis (34%, 56.6%) than CI (28.1%, 55.2%). Stapes surgery had a higher significant mean for speech reception threshold (62.6 dB) than CI (43.7 dB).

Published data about speech recognition scores with CI in far advanced otosclerosis patients ranged from 45 to 98%. Many studies showed better hearing results with CI than with stapes surgery [3, 25]. According to Calmel’s et al., 36% had a disyllabic word recognition at 70 dB and 45% have a percentage of satisfaction after stapes surgery [3]. Shea et al. reported that 42% of patients, who had no preoperative bone conduction thresholds, showed measurable thresholds after stapes surgery [29].

On comparing speech reception score in FAO after CI and stapes surgery, Bajin et al. found no significant difference [27]. lovato’s 2020 reported speech reception threshold 36 dB and word reception score 94% in FAO after CI. Glasscock et al., Calmels et al. described poor mean speech recognition after stapes surgery with of 33% and 54% respectively [3, 14]. According to Kabbara et al., 60% of stapes surgery group and 85% of CI group had successful outcome (Word Reception Score greater than 50%) [16]. Berrettini et al. and Calmels et al. stated that CI leads to statistically better mean speech recognition scores than stapes surgery [3, 30].

Meta-analysis of relevant studies showed that CI had significantly higher satisfaction rate (86.3%) in patients with far advanced otosclerosis than stapedectomy (69.5%). According to Bajin et al., many patients who had hearing problems for years tend to choose CI as the best route to restore hearing [27].

The results of our meta-analysis showed that the outcomes and complications of cochlear implantation and stapes surgery in FAO patients have different results. In most of them, CI is considered highly favorable and recommended procedure than stapes surgery, other results declared no significant difference in postoperative outcomes. Patients must receive adequate counseling regarding all the factors mentioned above and the decision must be made by surgeons and the informed patients.

## Conclusion

Both Stapes surgery and CI are reliable treatment options for FAO with close success rates. Statistics of CI are greater than stapes surgery and CI has a consistent improvement in audiometric outcomes in comparison to stapes surgery.
